# Characteristics of Treatment Naïve Chronic Hepatitis B in Bangladesh: Younger Populations are More Affected; HBeAg-negatives are  More Advanced

**DOI:** 10.4103/1319-3767.37796

**Published:** 2008-01

**Authors:** Shanhinul Alam, Nooruddin Ahmad, Golam Mustafa, Khorshed Alam, Mobin Khan

**Affiliations:** Department of Hepatology, Bangabandhu Sheikh Mujib Medical University, Shahbag, Dhaka, Bangladesh

**Keywords:** Age, Bangladesh, chronic hepatitis B

## Abstract

**Background/Aim:**

Bangladesh is a densely populated country with intermediate endemicity for chronic hepatitis B (CHB). The aim of the present study was to evaluate the biochemical, virological and histological character of CHB patients and to examine the relationship between these indices.

**Materials and Methods:**

One thousand and twenty-two patients of CHB fulfilled our inclusion criteria. Inclusion criteria were (1) HBsAg positive for at least 6 months, (2) HBeAg-positive or negative and (3) hepatitis B virus (HBV) DNA positive. Patients with detectable antibodies to human immunodeficiency virus (HIV), hepatitis Delta virus (HDV) or hepatitis C virus (HCV), with previous antiviral treatment, overt cirrhosis and hepatocellular carcinoma, were excluded. Of these, 191 patients were randomly selected for liver biopsy and were evaluated for analysis.

**Results:**

In the 191 patients, male to female ratio was 4.6:1; age distribution was 26.5 ± 8.5 (mean ± standard deviation) years. One hundred and seventy-eight (93.2%) patients were under 40 years. Sixty-eight (35.6%) patients were HBeAg-negative, had less DNA load, and were significantly older, more fibrotic and cirrhotic (*P* < 0.001). Correlation was not found between DNA level and histological activity. Histological activity was not correlated with ALT level in HBeAg-positive patients (*P* < 0.001).

**Conclusion:**

CHB affects the younger population in Bangladesh. HBeAg-positive CHB was associated with more fibrosis and cirrhosis. Serum HBV DNA levels do not correlate with the severity of histological lesions in all patients. Evaluation by liver biopsy remains gold standard for taking decision of treatment.

An estimated 350 million persons are chronically infected with hepatitis B virus (HBV) worldwide.[1] Approximately 15–40% may develop serious sequelae, including cirrhosis and hepatocellular carcinoma.[2] Patients with hepatic inflammation and fibrosis are at the highest risk of complications.[3–7] With a population of 150 million, Bangladesh has a high HBsAg positivity in adults (7.3–7.5%).[8,9] HBeAg-negative is an important form of chronic hepatitis B (CHB).[10,11] This form of CHB is being increasingly reported worldwide.[12–19] The aim of this study was to evaluate the different characteristics of CHB patients and to examine the relationship between them.

## MATERIALS AND METHODS

This cross-sectional study was conducted during the period between January 2001 and March 2007 at the Department of Hepatology of Bangabandhu Sheikh Mujib Medical University (BSMMU) Hospital, Dhaka, Bangladesh. During this period, Patients who were included in this study had (1) HBsAg positive for at least 6 months, (2) HBeAg-positive or negative and (3) HBV DNA positive. Patients with human immunodeficiency virus (HIV), hepatitis Delta virus (HDV) or hepatitis C virus (HCV), those who previously had antiviral treatment as well as those with hepatocellular carcinoma were excluded. Patients with overt cirrhosis and autoimmune hepatitis were also excluded from the study. Schistosomiasis is very uncommon in Bangladesh, and yet was excluded from the study. 3141 patients of CHB were treated in the center. Out of them, 1022 patients met our inclusion criteria; of these patients, with systematic random sampling and sample interval of 5, we had conducted liver biopsy in 191 patients; the rest of the patients refused to undergo liver biopsy. Biopsy was performed with an interval of 0–7 days from other investigations. Before liver biopsy, informed written consent was taken from every patient. The protocol was reviewed and permitted by the departmental ethical review committee. Liver histology was assessed by a pathologist who was uninformed of the results of liver biochemistry and HBV DNA levels. The histology was graded by the histologic activity index (HAI) according to the criteria of Knodell *et al*.[20] The total HAI score comprises two major components, namely necroinflammation and fibrosis, which includes piecemeal necrosis, lobular necrosis and inflammation, portal inflammation and fibrosis. Different characteristics in patients were assessed, including age, sex and source of infection. Laboratory tests included ALT, AST, HBsAg, anti-HBs and anti-HBc antibodies, HBeAg and anti-HBe antibodies. HBV serological markers were detected using enzyme-linked immuno absorbent assays (Abbott Laboratories, North Chicago, IL, USA). Serum HBV DNA was determined by a solution hybridization assay based on hybrid-capture (Digen Hybrid-Capture; Murex Diagnostics, Dartlord, UK) with the detection range of 1.42 × 10^5^ to 1.7 × 10^9^ copies/ml or a target-amplification assay based on competitive polymerase chain reaction (Amplicor HBV Monitor™; Roche Molecular Systems, Pleasanton, CA, USA) with the detection range of 300 to 10^6^ copies/ml, which was increased with dilution.

### Data analysis

Results were expressed as mean ± standard deviation (SD) or percentage. Independent *t* test was used to compare continuous variables. The Chi-square test was used to compare categorical data. One-way ANOVA with post hoc test was performed to compare more than two means. Pearson correlation was performed for correlation analysis. Statistical analysis was performed after log_10_ conversion when distribution was not normal (DNA and ALT). Statistical analysis was performed using SPSS 10.0 software (1999, Chicago, USA), and a *P*-value of <0.05 was considered as significant.

## RESULTS

One hundred and ninety-one patients were included in the study. The male-to-female ratio was 4.6:1. The age range was 14–55 years (mean ± SD: 26.5 ± 8.5 years). Overall DNA load was log_10_ (8.35 ± 8.63) copies/ml; histological activity was 6.8 ± 3.2; ALT level was (93.4 ± 144.3) U/L; and AST was 59.5 ± 44 (mean ± SD). Forty (20.9%), 89 (46.6%), 54 (28.3%) and 8 (4.2%) patients had minimal, mild, moderate and severe necroinflammatory activity, respectively. Out of 40 patients with minimal histological activity, 19 patients were HBeAg positive, 12 patients had no fibrotic activity, 22 had normal ALT, and 15 had high DNA level; these 15 were possible immune-tolerant CHB. One hundred and seventy-eight (93.2%) patients were under 40 years. We categorized the patients in groups of <20 years of age, 21–30 years, 31–40 years, and >40 years. The ALT, necroinflammatory activity and fibrosis had no significant difference across different age groups [Table 1]. Age had inverse relationship with DNA level. Most of the patients (84.8%) had no history of jaundice, hospitalization, surgery, dental procedure or blood transfusion. None of the patients gave a history of intravenous drug abuse or alcohol intake.

**Table 1:** Biochemical, histological and virological character at different age groups

Age (years)	ALT U/L	Necroinflammatory activity	Fibrosis	DNA log copies/ml
≤20 (*n* = 53)	83.0 ± 57.2	6.9 ± 3.4	1.9 ± 1.3	7.3 ± 1.5
21-30 (*n* = 90)	72.3 ± 63.9	6.2 ± 3.4	1.4 ± 1.1	7.0 ± 1.2
31-40 (*n* = 35)	69.1 ± 58.5	7.0 ± 3.0	1.8 ± 1.1	6.2 ± 1.4
>40 (*n* = 13)	63.6 ± 41.0	5.8 ± 2.8	1.6 ± 1.3	5.8 ± 1.6

### Characteristics of HBeAg-positive and HBeAg-negative patients

The overall proportion of HBeAg-negative CHB was 68 (35.6%). HBeAg-negative patients had less DNA load and were significantly older and more fibrotic than HBeAg-positive patients. However, they showed insignificant difference in necroinflammatory activity and ALT level against HBeAg-positive patients. Cirrhosis was more common in HBeAg-negative patients [Table 2]. Sex difference was insignificant between HBeAg-positive and HBeAg negative patients.

**Table 2:** Comparison of different characteristics of HBeAg-positive and HBeAg-negative patients

Characteristics	HBeAg-positive (*n* = 123)	HBeAg-negative (*n* = 68)	*P*-value
Age (years)	23.8 ± 7.0	30.4 ± 9.6	<0.001
Sex (male) (%)	83.7	80.0	0.557
Unknown source of infection (%)	92.2	78.8	
Serum ALT level U/L	100.1 ± 170.5	81.3 ± 77.4	0.297
Serum AST level U/L	56.4 ± 36.4	65.5 ± 110.7	0.578
Serum HBV DNA (log copies/ml)	7.6 ± 1.3	5.8 ± 1.4	<0.001
**Liver histology**			
Activity score	6.8 ± 3.0	6.7 ± 3.6	0.89
Fibrosis score	1.4 ± 1.1	1.92 ± 1.2	<0.001
Cirrhosis (%)	1.7	8	<0.001

### Correlation between HBV DNA levels and histological activity

Correlation was not found between DNA level and histological activity of both HBeAg-positive and HBeAg negative patients. It was not also correlated with fibrotic activity of all groups of patients. Histological changes and ALT level did not significantly differ with different levels of DNA [Table 3]. DNA level ≥10^7^ copies/ml was associated with HAI of 6.8 ± 3.1 and DNA level <10^4^ copies/ml had HAI of 7.7 ± 3.7. Eight patients had DNA level below the recommended level of treatment, 6 of them from HBeAg negative group and 2 of them from the HBeAg-positive group and all of them had significant histological activity. Six patients of HBeAg-negative group with DNA level below 10,000 copies/ml had histological activity of 8.5 ± 3.3. There was no significant difference in DNA level (*P* = 0.339) between minimal to mild and moderate to severe histological activity (cut-off point of HAI was 8)

**Table 3:** Histological and biochemical changes at different DNA level

DNA log copies/ml	3-<4 (*n* = 7)	4-<5 (*n* = 13)	5-<6 (*n* = 42)	6-<7 (*n* = 25)	≥7 (*n* = 103)
Necroinflammatory activity	7.7 ± 3.7	6.5 ± 3.8	5.8 ± 3.0	7.8 ± 3.5	6.8 ± 3.1
Fibrosis	1.8 ± 1.3	1.6 ± 1.2	1.6 ± 1.1	2.1 ± 1.2	1.5 ± 1.8
ALT	48.2 ± 28.2	62.1 ± 23.3	113.5 ± 282.2	98.2 ± 80.2	90.0 ± 66.3

### Relationship between ALT level and histological activity

Histological activity was positively correlated with serum ALT level inHBeAg-negative and overall patients (*P* < 0.001) but not in HBeAg-positive patients. In this study, a significant number of patients (44, 23%), equally from both HBeAg-positive and negative groups, had ALT level below the normal limit, who had significant histological activity (5.3 ± 2.8). One hundred and forty-four (75.4%) patients had elevated ALT level, and it was positively correlated with histological activity. Patients with ALT <1ULN, <2ULN, <3ULN, <4ULN, <5ULN and >5ULN had histological activity of 4.8 ± 2.7, 6.9 ± 3.0, 7.3 ± 3.2, 8.5 ± 3.3, 7.0 ± 2.8 and 9.3 ± 2.7, and DNA levels of 6.4 ± 1.6, 7.1 ± 1.6, 7.1 ± 1.6, 7.5 ± 1.1, 6.2 and 7.3 ± 1.3 log copies/ml, respectively.

### Fibrosis

Fibrosis was more in the male sex, HBeAg-negative cases and in the elderly. There was no significant correlation with DNA level, route of exposure and smoking.

## DISCUSSION

Bangladesh is an intermediate endemic country for hepatitis B, with a huge burden of CHB patients in its population. This report is first of its kind from Bangladesh on the characteristics of CHB patients. It is representative of 1022 CHB patients. BSMMU is the only tertiary care referral center for management of CHB patients in Bangladesh, where patients from all over the country are being referred to. In this study, 93.2% CHB patients were under 40 years, being younger than those reported from European populations.[21] It may be due to early exposure of the subjects to HBV during perinatal period like in other Asian countries.[22] Most of these patients were not in the immune-tolerant phase as evidenced by necroinflammatory activity, DNA load and ALT level. The conversion to immune-active phase was earlier but not completely dissimilar to other studies where it occurred after 10–30 years.[23] In this study, the HBeAg-positive CHB subjects were 23.8 ± 7.0 years old and HBeAg-negative subjects were 30.4 ± 9.6 years old, and male sex was predominant in both the groups. In this series, relatively younger subjects were in the immune-active phase against that reported in other studies, where HBeAg-positive CHB subjects were 24–36 years old at initial presentation. Sexual differences were similar in those studies.[5,10,11,24–26] Liver histology showed mild activity in 46.6% and moderate to severe activity in 32.5% of the patients in this study. In other studies, mild activity was described in 24–42%, and moderate to severe activity in 44–63%.[15,24–29] These differences could be related to the younger age of the patients in our study.

In CHB, HBeAg-negative is prevalent in 80–90% in Italy,[16,30] Greece[17,31] and Asia. In France, a prevalence of 22.1% was reported in 1994 in a population of 276 consecutive patients with CHB. Recently, prevalence increased in America,[32] northern European countries[33] and France.[21] In this series, the prevalence of HBeAg-negative CHB was 35.6%. It was in-between the two previous reports from Hong Kong and Korea, where prevalence was 69% and 19.6%, respectively.[18,19]

As reported in other studies, age was significantly higher in HBeAg-negative patients than in HBeAg-positive patients, but unknown source of infection was higher in HBeAg-positive patients, which is different from other studies.[13–19,34] ALT and HBV DNA levels were significantly lower in HBeAg-negative subjects. These results are in accordance with recent studies, where HBV DNA levels were lower in a majority of HBeAg-negative CHB patients. We know that HBeAg-negative patients have an erratic pattern of ALT changes, and HBV DNA level can be observed with frequent fluctuations.[35,36] Histological lesions were more severe in HBeAg-negative patients than in HBeAg-positive patients. Necroinflammatory activity was almost similar in both groups, but fibrotic activity was more in HBeAg-negative group.

We found no relationship between HBV DNA levels and liver histology in terms of necroinflammation and fibrosis in CHB patients. It may be that, for patients in immune-tolerant phase, HBV DNA is high since immune-mediated injury has not commenced yet. On the other hand, in immuno-clearance phase, the severity of liver histology will markedly increase by immune-mediated response and will lead to low viremic condition. This finding was in agreement with other studies in case of HBeAg-positive patients but different for HBeAg-negative patients.[37–40] Our study was limited by the lack of genotyping for HBV, and this aspect needs to be evaluated further in future studies.

Indeed, the proportion of cirrhosis was 1.7% in e antigen-positive group and 8% in HBeAg-negative group. Fibrosis was more in elderly, HBeAg-negative and male patients. These results are in accordance with many other published studies, where old age and male sex are important factors associated with the progression of CHB. This may be due to the mechanisms of fibrosis in hepatitis B as reported in other chronic liver diseases and especially chronic hepatitis C related to host-related factors.[4,41–45]

In conclusion, CHB affects the younger population of Bangladesh, and may lead to the development of cirrhosis and hepatocellular carcinoma at younger ages. HBeAg-negative patients account for one-third of cases of CHB. It was associated with more severe histological lesions than those of HBeAg-positive patients. This study showed that HBV DNA levels have no clear relationship with severity of histological lesions. The evaluation of CHB by liver biopsy remains the gold standard for implementing treatment decisions.
